# Promoting Pollution-Free Routes in Smart Cities Using Air Quality Sensor Networks

**DOI:** 10.3390/s18082507

**Published:** 2018-08-01

**Authors:** Francisco Ramos, Sergio Trilles, Andrés Muñoz, Joaquín Huerta

**Affiliations:** Institute of New Imaging Technologies, Universitat Jaume I, Av. Vicente Sos Baynat s/n, 12071 Castellón de la Plana, Spain; jromero@uji.es (F.R.); zuluaga@uji.es (A.M.); huerta@uji.es (J.H.)

**Keywords:** air quality indices, air quality sensors network, spatial interpolation, routing service

## Abstract

Nowadays, citizens have a huge concern about the quality of life in their cities, especially regarding the level of pollution. Air quality level is of great importance, not only to plan our activities but also to take precautionary measures for our health. All levels of governments are concerned about it and have built their indexes to measure the air quality level in their countries, regions or cities. Taking into account the existing sensor infrastructure within smart cities, it makes possible to evaluate these indices and to know anywhere the level of pollution in real-time. In this scenario, the main objective of the current work is to foster citizens’ awareness about pollution by offering pollution-free routes. To achieve this goal, a technology-agnostic methodology is presented, which allows for creating pollution-free routes across cities depending on the level of pollution in each zone. The current work includes an extensive study of existing air quality indices, and proposes and carries forward to deployment of the defined methodology in a big city, such as Madrid (Spain).

## 1. Introduction

Smart cities use information technologies to improve on the performance and quality of urban services, to decrease costs and optimize resources, and more so they involve citizens to participate actively in such activities [1]. Among the various areas developed in such a context, we underline public health, in which this study is focused on. In particular, many efforts have been carried out to improve air quality in cities which has led to the establishment of different measures for air pollution.

In recent years, air pollution control has demonstrated to have a positive impact on public health [2]. A control measure taken by governments or local administrations involves using specific sensors distributed over a wide area usually named air pollution sensors, which can detect different levels of air pollution in a particular location.

Deployment of these sensors is slightly growing up with smart cities and Internet of Things (IoT) [1] initiatives, which enables us to obtain access to their data. However, the information usually provided to the users is one-dimensional space-based, mainly corresponding to a determined and fixed latitude and longitude where the sensor is located.

Many works have been done to publish or generate two-dimensional space data from those types of sensors, among them, we underline the ones using interpolation methods that use spatial analysis by applying statistical theory and techniques in order to model spatially referenced data.

There are some works that their objective is to monitor environmental conditions inside a smart building [3,4] where observations are managed at spatial points. In our context, wide metropolitan areas, pollutant concentrations are types of data that can be represented by surfaces where each raster cell represents a measurement such as a cell’s relationship to a fixed location or specific concentration level. Due to the impracticability of obtaining values for each cell in a raster, sample points are used to derive the intermediate values using interpolation methods. This ability to create surfaces from sample data of air pollution sensors makes spatial interpolation both powerful and useful for this work.

In disseminating pollution information, several government bodies and industries publish pollutant concentration levels on their websites or mobile applications. The published report can be in the form of pollutant concentrations or scaled concentrations based on particular air quality or air pollution index. Some of these indices provide health-related recommendations to the general public or specific groups of people for the different levels of pollutant concentrations.

In this context, we present a technology-agnostic methodology, which consists of the use of air quality networks to create pollution-free routes across cities. At first, an Air Quality Index (AQI) is deployed based on a review study to achieve this goal. This index will be used to categorize air quality observations, and, in a second step, this categorization will be interpolated to create a final map service with different zones. In the last step, this map service will be used to trace routes avoiding high polluted areas.

In summary, the main contributions of this work are: (1) a technology-agnostic methodology to connect in real time to air quality sensor networks to create pollutant-free routes inside cities; (2) perform a broad analysis of existing AQIs; and (3) propose and carry forward a development of the defined methodology in a big city, such as Madrid.

The rest of the paper is organized as follows: Section 2 presents the background and fundamentals used as a basis of our work: a comparative study of existing air quality indices and interpolation methods for air pollution contexts. Section 3 presents an overview of the methodology created to create pollutant-free routes. Section 4 details how a proof of concept proposed in Madrid has been developed to test and validate the technology agnostic methodology presented in the previous section. Finally, in Section 5, conclusions and future work are presented.

## 2. Background

The Background part begins in Section 2.1 by detailing the most essential air quality indices and presents a comparative study of the reviewed indices.

### 2.1. Comparative Study of Air Quality Indices

This study compares some of the formulated indices from governmental bodies and the research community based on their definitions and calculations, categories and ranges considered, used symbology in their representations, general health recommendations to the public and specific groups of people, the effect of multi-pollutants and concentration measurement location variations. In the air quality and air pollution fields, some pollutants considered are Carbon monoxide (CO), Nitrogen Dioxide (NO${}_{2}$), Ozone (O${}_{3}$), PM10, PM2.5 and Sulfur Dioxide (SO${}_{2}$). In this section, we have selected and described some of the implemented indices by government departments and industry.

#### 2.1.1. The United States Environmental Protection Agency (EPA) Air Quality Index (AQI)

The US EPA AQI categorizes air quality in six categories of Good with range (0–50), Moderate with range (51–100), Unhealthy for Sensitive Groups with range (101–150), Unhealthy with range (151–200), Very Unhealthy with range (201–300) and hazardous with range (301–500). These categories increase with increasing effect on human health and are assigned standard colors for easier identification and reporting (US EPA, 2013). This AQI is defined for pollutants of O${}_{3}$, PM2.5, PM10, CO, NO${}_{2}$ and SO${}_{2}$.

#### 2.1.2. The Canada Air Quality Health Index (AQHI)

The AQHI communicates the air quality health-related risks on a scale of 1 to 10+ with four categories of health risks as Low Health Risk (1–3), Moderate Health Risk (4–6), High Health Risk (7–10), or Very High Health Risk (10+). A color scheme of light blue for lower values of the index to brown for higher values of the index is used in the index communication. It also gives the health messages for both the population at risk and the general population. AQHI uses the relative risks of a combination of pollutants of O${}_{3}$, PM2.5 and NO${}_{2}$ to determine the final index.

#### 2.1.3. Common Air Quality Index (CAQI)

CAQI defines five classes with appropriate ranges of Very Low (0–25), Low (25–50), Medium (50–75), High (75–100) and Very High (above 100). It considers pollutants of O${}_{3}$, PM10, CO, NO${}_{2}$, SO${}_{2}$ and PM2.5 [5,6]. CAQI uses the concept of core pollutants that allow for the calculation of the index. For the roadside index, the core pollutants are NO${}_{2}$ and PM10 with CO and PM2.5 as auxiliary pollutants, while, for the city background index NO${}_{2}$, PM10 and O${}_{3}$ are core pollutants with CO, SO${}_{2}$ and PM2.5 are auxiliary pollutants. The final index is given as the highest sub-index of the considered pollutants [5,7].

#### 2.1.4. Daily Air Quality Index (DAQI)

DAQI is defined on a scale of 1 to 10 with color coding and categorized into four bands of Low (1–3), Moderate (4–6), High (7–9) and Very High (10). Currently, the DAQI uses the pollutants of O${}_{3}$, NO${}_{2}$, SO${}_{2}$, PM2.5 and PM10 in the calculation of the index. The overall index is given by the highest pollutant concentration of the considered pollutants [8].

#### 2.1.5. Ireland Air Quality Index

This index uses a scale with the defined color coding of 1 to 10. It is categorized into four bands of Good (1–3), Fair (4–6), Poor (7–9) and very poor (10) [9]. These bands correspond to the UK DAQI bands of Low, Moderate, High and Very High, respectively. The other information like health messages and interpretation of the index is the same. Like the DAQI, AQIH considers five pollutants of O${}_{3}$, NO${}_{2}$, SO${}_{2}$, PM2.5 and PM10. The final index is given by the worst index of the separately calculated indices of the considered pollutant concentrations.

#### 2.1.6. Spain, Madrid Air Quality Index

Madrid City Council provides information to its community using an AQI defined by a scale of 0 to >150 with four categories of “Buena” (0–50), “Admisible” (51–100), “Deficiente” (101–150) and “Mala” (>150). The City council uses the pollutants of PM10, SO${}_{2}$, NO${}_{2}$, CO and O${}_{3}$. Sub-indices are calculated for the considered pollutants, and the final index is the worst sub-index of the pollutant concentrations [10].

#### 2.1.7. France Air Quality Index, ATMO Index

The ATMO Index is the AQI used in major cities in France that have a population of more than 100,000 inhabitants. The index is represented by a giraffe and is based on a scale of 1 to 10 ranging from very good to very bad and with three colored bands of Green (1–4), Orange (5–7) and Red (8–10). ATMO Index considers the pollutants of SO${}_{2}$, NO${}_{2}$, O${}_{3}$, PM2.5 and PM10. Sub-indices are calculated for the four pollutant concentrations, and the final aggregated index is the highest sub-index calculated from the pollutant concentrations [11].

Each pollutant has defined limit values for the scale ranges upon which pollutant concentrations are compared to determine the pollutant sub-index.

#### 2.1.8. Singapore Pollutant Standards Index (PSI)

PSI is categorized into five categories of Good (0–50), Moderate (51–100), Unhealthy (101–200), Very Unhealthy (201–300) and Hazardous (301–500) [12]. PSI is based on six pollutants of PM2.5, PM10, SO${}_{2}$, CO, O${}_{3}$ and NO${}_{2}$. The sub-indices of all the considered pollutants are calculated, and the final index is the highest sub-index of the pollutant concentrations.

#### 2.1.9. Researchers Work on Air Quality Index Representation

In this subsection, we specifically selected five formulated and published indices from the research community that relate to health, the effect of multi-pollutants on health and spatial variability of pollutant measurement locations.

To account for multi-pollutant short-term health effects of exposures on the final index, Ref. [13] formulated an Air Pollution Index (API) which gives the final index as the summation of the normalized pollutant sub-indices. The index depends on the relative risk of daily mortality associated with the common pollutants of PM2.5, PM10, SO${}_{2}$, NO${}_{2}$, O${}_{3}$ and CO. It has a scale of 1 to 10 with four color-coded categories and the associated increase in mortality risks. The categories are low (1–3), moderate (4–6), high (7–9) and very high (10).

Based on time series analysis of air pollution and mortality in Canadian cities, Ref. [14] proposed an Air Quality Health Index (AQHI). To cater for multi-pollutant effects and different seasons, they carried out analysis for pollutant combinations using single and multi-pollutant models and for different seasons. They based on the association of CO, NO${}_{2}$, O${}_{3}$, PM2.5 or PM10 and SO${}_{2}$ pollutants to develop the index. The index is defined by four different scenarios using PM2.5, PM10, warm and cold seasons for the case of PM2.5. The index is on a scale of 0 to 10+ with color-coded categories of Low risk (0–3), Moderate risk (4–6), High risk (7–10) and Very high risk (above 10). It provides health-related messages to the population at risk and the general population.

Using the pollutants of CO, SO${}_{2}$, NO${}_{2}$, O${}_{3}$ and PM10, Ref. [15] developed the aggregate AQI for Athens, Greece. To cater for multi-pollutant effects, they adopted an aggregate function to compute the overall index of the city. They compared their results with the modified United States Environmental Protection Agency (US EPA) AQI using the European pollutant standard limits and found that the modified USEPA predicted higher values than the developed aggregate AQI.

In studying multi-pollutant effects and relative risks of short-term exposure to pollutants, Ref. [16] developed the Aggregate Risk Index (ARI). It is based on the exposure-response relationship and relative risk of established effects to assess the pollutants’ additive effects. They used relative risk functions data and particular sets of relative risks for associated health risk endpoints to derive the index. ARI considers the relative risks of SO${}_{2}$, NO${}_{2}$, PM2.5, PM10 and O${}_{3}$ pollutants. In catering for the multi-pollutant effects, the final index is the summation of individually calculated risk indices. The index is defined from 0 to 10 with the risk values used to derive the breakpoints. The index is categorized into Low (1–3), Moderate (4–6), High (7–9) and Very high (10) with appropriate information about the excess relative risk of mortality or morbidity.

In considering the additive effects resulting from multi-pollutants and the impact of measuring pollutants over different geographical locations, Ref. [17] developed a daily air Pollution Index (PI) modified out of the USEPA AQI. The developed index uses European limit values in its computation. The index uses the common pollutants of CO, NO${}_{2}$, PM10, SO${}_{2}$ and O${}_{3}$ in its computation. The index is defined on a scale of 0 to 100 and defines five categories represented by clouds of Good quality (25), Low pollution (50), Moderate pollution (70), Unhealthy for sensitive groups (85) and Unhealthy (100). PI considers the sum of ratios of daily reference concentrations of pollutants and their bottom breakpoint concentration values to cater for multi-pollutants’ additive effects on human health and introduces weights for geographical location variability of sensor measured pollutants concentrations.

#### 2.1.10. Summary of the Reviewed Indices

The reviewed indices share and differ in some features of their formulations and representations. We have compared them concerning pollutants considered, the number and ranges of categories, the symbolization, and graphical representation, the health recommendations for the categories, the effect of multi-pollutants and the spatial variability of concentration measurement locations. Table 1 shows the comparison of governments’ departments implemented indices and some of the indices developed by the research community.

Table 1 shows that most indices share the pollutants composition with O${}_{3}$, PM10 and NO${}_{2}$ pollutants, which are common to all indices. They vary in the formulation of categories ranging from 3 to 6, four being the most used number of categories. Apart from two indices with undefined color symbology, all the other indices are color-coded along their respective categories. Most indices also give general and specific health recommendations to the public. The indices, from the research community, considered more the effect of multi-pollutants compared to the researched or implemented indices by government bodies. Finally, most indices do not consider the location variability of pollutant measurements.

## 3. A Technology-Agnostic Methodology to Trace Pollution-Free Routes

**Figure 1:**
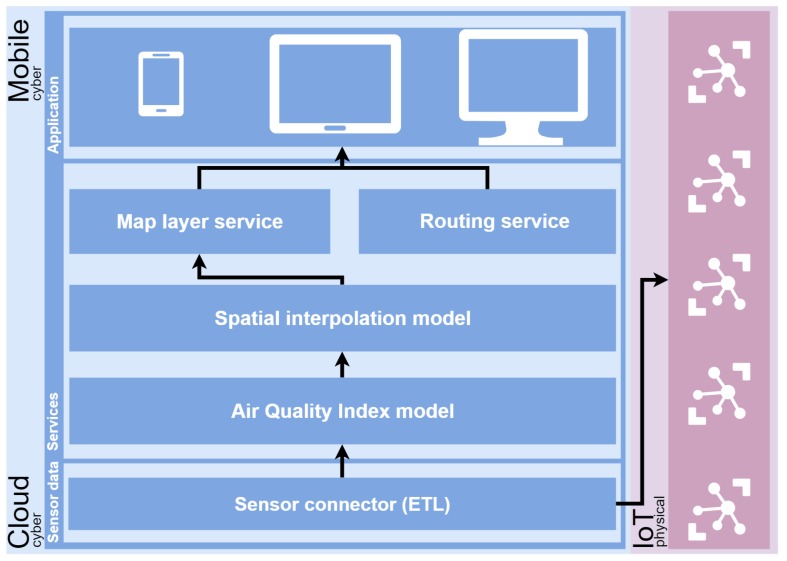
A representation of the technology-agnostic methodology to trace pollution-free routes; the physical environment is shown in purple; the cyber environment is represented in blue and is divided into three layers: data, service and application layers.

As we commented in Section 1, the primary goal of our work is to propose a technology-agnostic methodology to provide a framework to calculate pollution-free routes, that is, routes where pollution is the main component that is taken into account. Thus, we propose a structure based on three layers (data, services, and applications) and two different environments (Figure 1).

The methodology contemplates and works on two different environments, *physical* and *cyber*. The first of them focuses on the devices of the IoT, which have direct access to the Internet. These devices are the air quality stations and are used as an input of the models to generate the pollution-free routes.

Moving to *cyber* site, a three layer approach is defined. It is composed by *Data layer*, *Service layer* and *Application layer*. Each of them is detailed as follows.

The *Data layer* connects with the physical environment in order to obtain the observations from an air quality network; it is possible using an ad hoc connector with each station. It is used as a bridge between the *data layer* and the IoT devices (air quality stations), following the same strategy detailed in [18]. A collection of connectors can be defined to connect with different networks. The definition of standard connectors is also contemplated to connect with the networks that are available with these standards, such as OGC Sensor Observation Service (SOS) [19] or SensorThings API [20]. In addition, these connectors have the objective to create a set of well-formatted measurements ready to be used by the next layer (*Service layer*).

The *Service layer* is composed of several components, and all processes and services are hosted. The first component is the AQI model, which, according to the information received from the sensor connector, can build an index defined by some pollutants. As described in Section 2.1, there exist a wide range of formulated indices and an in-depth study becomes necessary to establish the AQI to be used in the context or environment where this methodology is applied. Thus, the output of this component will serve as the source for the next component: the Spatial Interpolation Model, which is responsible for generating a 2D surface by estimating the values of unsampled points from the values of sampled points provided by the component mentioned above.

Finally, on the top of the *Service layer*, we have the map layer service and the routing service; both services allow for performing different operations in the *Application layer* such as obtaining a route minimizing the step through the areas with high pollution or receiving an alert in a mobile device when entering a zone with high pollution. The Application layer can support any kind of client to visualize the final results, such as web, mobile or desktop.

## 4. A Proof of Concept in Madrid

From the previous section, we propose a proof of concept to develop and validate the detailed technology-agnostic methodology. To archive this, we selected Madrid as a scenario to test our approach; the main reason is that there is an available air quality sensor network.

In this section, we are dividing into four different subsections. First, of these four methods, we introduce the use case itself in Section 4.1 and how we can connect with the air quality sensor network from Madrid (Section 4.2). The third subsection (Section 4.3) details a comparative study to select a suitable AQI and Section 4.4 proposes a formulation of the selected AQI. Section 4.5 determines a selected spatial interpolation to analyze observations from Madrid sensor network. The next step is to serve the results from the spatial interpolation thought a map processing service (Section 4.6). Section 4.7 shows the routing service to avoid pollutant zones. Finally, Section 4.8 exhibits a web-application to trace routes from the final user side.

### 4.1. Madrid Air Quality Network Use Case

The city council of Madrid has a network of 24 sensor stations deployed to measure different pollutants in Madrid city to enable pollution and air quality monitoring and management. The city council has been monitoring air quality since 1968 using a standard network and later on a set of automatic network stations since 1978. Due to the studies, developments, and legislation about air quality, the city council has continued to refine and develop this network to accommodate the developments.

The sensor stations’ network continually measures the pollutants of Sulfur dioxide, Carbon monoxide, Nitrogen monoxide, Nitrogen dioxide, PM2.5, PM10, Nitrogen oxides, Ozone, Toluene, Benzene, Ethylbenzene, Metaxylene, Paraxylene, Orthoxylene, Total hydrocarbons, Methane or Non- methane hydrocarbons.

The stations in this sensor network are categorised into three different classes: *Tráfico*—traffic, *Urbana de fondo*—Urban background and *Suburbana*—suburban. At each station class, several pollutants are measured, but the combinations of measurements at each station are quite different. The *Tráfico* sensor stations are mainly located along the road network and close to the city center for detecting pollution caused by emissions on the road network while the other two types are located mainly outside the area covered by the *tráfico* sensor stations. The *Urbana de fondo* sensors mainly represent the exposure of the general urban population while the *Suburbana* sensors are located in the city outskirts at locations of high ozone levels.

Stations are identified by station codes and pollutants identified by parameter codes for the pollutants measured at each station:

- *Tráfico*—traffic stations. These are stations 4 (Pza. de España), 8 (Escuelas Aguirre), 11 (Avda. Ramón y Cajal), 36 (Moratalaz), 38 (Cuatro Caminos), 39 (Barrio del Pilar), 48 (Castellana), 50 (Plaza Castilla) and 56 (Pza. Fernández Ladreda).
- *Urbana de fondo*—Urban background. These are stations 16 (Arturo Soria), 17 (Villaverde), 18 (Farolillo), 27 (Barajas Pueblo), 35 (Pza. del Carmen), 40 (Vallecas), 47 (Mendez Alvaro), 49 (Parque del Retiro), 54 (Ensanche de Vallecas), 55 (Urb. Embajada), 57 (Sanchinarro) and 60 (Tres Olivos Plaza).
- *Suburbana*—suburban. These are stations 24 (Casa de Campo), 58 (El Pardo) and 59 (Juan Carlos I).

**Figure 2:**
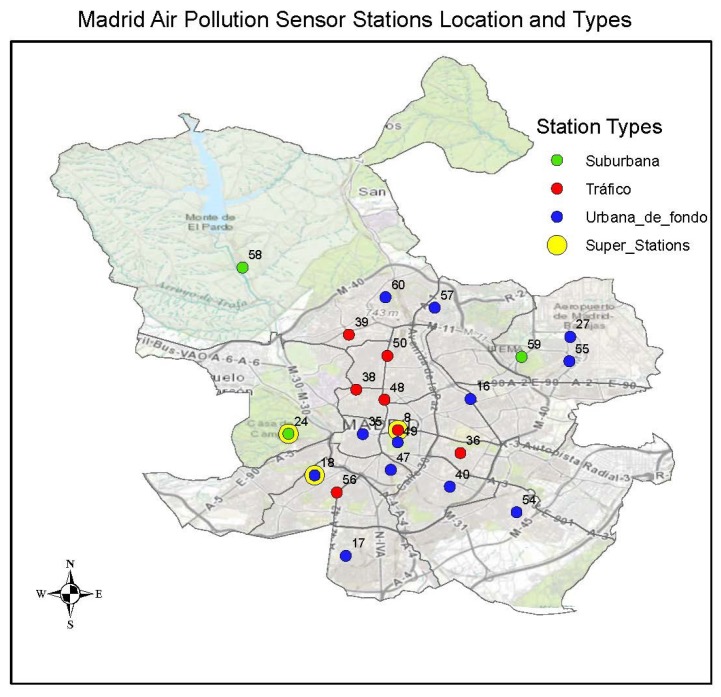
Map with the air quality stations locations and types.

Within this network, there are three *full* stations and these measure most of the network pollutant components and consider all the types of *tráfico*, *urbano de fondo* and *suburbana*. These stations are the number 18 (Farolillo (without PM2.5)), 24 (*Casa de Campo*) and 8 (Escuelas Aguirre). The location of each sensor is shown in Figure 2.

### 4.2. Connecting to Madrid Air Quality Sensors Network

The Madrid city council publishes data from monitoring sensor stations on an hourly rate and also provides historical data. The data is encoded in different file formats like eXtensible Markup Language (XML), Comma-Separated Values (CSV) or text file (TXT) and contains pollutant concentration measurements. For the hourly data, each station file contains: a station code, sensor codes and dates at which the values were recorded. For historical data, each file contains, for all stations, a month of their daily hourly values. The data are published on Madrid’s open data portal [21].

At a single monitoring station, different sensor pollutants’ measurements are collected and published with no geographic location. Since sensor station location is essential for spatial interpolation, there was a need to incorporate these locations into sensor stations’ measurements. The Madrid city council provides also a description and location (latitude, longitude, and altitude) of their monitoring stations’ network. These coordinates are transformed into a projected coordinate system (ETRS89UTMzone30N) to support structural modelling and parameter control.

From a technical point of view, an ETL process encoded in a script is created to retrieve the sensor data from the open data portal. This module retrieves real-time and historical data, checks the data, and extracts only the pollutants and concentration values which are of interest in the study. It will be extended to add other defined steps (index calculation, interpolation, …) in the workflow. This script will be launched using a task scheduler manager depending on the sensor network refresh that, in our use case, is every hour.

### 4.3. Selecting an Applicable AQI

To select an applicable AQI for our study, we have related the available Madrid sensor data with some of the reviewed AQIs for the study. We base this relation on the indices’ definitions (Section 2.1) with the pollutant combinations in their formulations. From the discussed AQIs (Section 2.1), the indices of AQHI (Canada) preferred for its linkage to health, CAQI (European), DAQI (UK Defra), Madrid Spain and ATMO (France) preferred for their formulation with the limit values of the European Union. We have been compared with the available data from the sensor stations and this comparison is given in Table 2.

From Table 2, it can be seen that using the AQIs with the pollutant combinations considered during their formulations presents a challenge in interpolation as most of the AQIs are presented as ad hoc sensor stations that fulfill such pollutant combinations. For the AQI used by the Madrid city council, it is the three *full* stations that accommodate this pollutant combination though these stations are close to each other and would hardly represent the air quality situation of all of Madrid.

With the primary sources of pollution in Madrid being Nitrogen Dioxide mainly due to heavy traffic, ozone and rPM [22], a couple of pollutant combinations were suggested to support interpolation of the data from the available sensor stations. Several scenarios of pollutant combination have been related and analyzed with some of the reviewed AQIs in getting the optimum scenario to serve the purpose of the study.

**Scenario** **1.** *Considering the AQHI established by Stieb et al. [14]*

The AQHI defined by [14] considers either PM10 or PM2.5. Opting to use the AQHI defined by PM10 facilitates four candidate sensor stations from which we can interpolate the data. However, consideration of the AQHI that uses PM2.5 facilitates two candidate sensor stations from which data can be interpolated. The other shortcoming of this index for this study is that it was formulated with the concentration response coefficients derived from Canadian mortality data and would not represent the situation in this study area.

**Scenario** **2.** *Considering the CAQI’s Roadside index without carbon dioxide as one of the auxiliary pollutants*

Using a combination of Nitrogen Dioxide and PM10 as core pollutants with PM2.5 as an auxiliary pollutant facilitates six sensor stations from which data can be interpolated. Most of these stations are near the city center of the type *Tráfico*—traffic stations. This scenario facilitates prediction of the central part of the city and not the city as a whole.

**Scenario** **3.** *Considering the CAQI’s City Background index without PM10 a core pollutant and without PM2.5 as an auxiliary pollutant*

This scenario facilitates four sensor stations to support interpolation though these stations are close to each other and may not give a better interpolation and representation of the entire city.

**Scenario** **4.** *Considering the CAQI’s City Background index without PM10 as a core pollutant and without auxiliary pollutants*

In this scenario, where we only consider a combination of Nitrogen Dioxide and Ozone, it facilitates 14 sensor stations from which to interpolate the data. The challenge with this scenario is that we would neglect both PM2.5 and PM10, which are some of the pollutants of concern in the study area.

**Figure 3:**
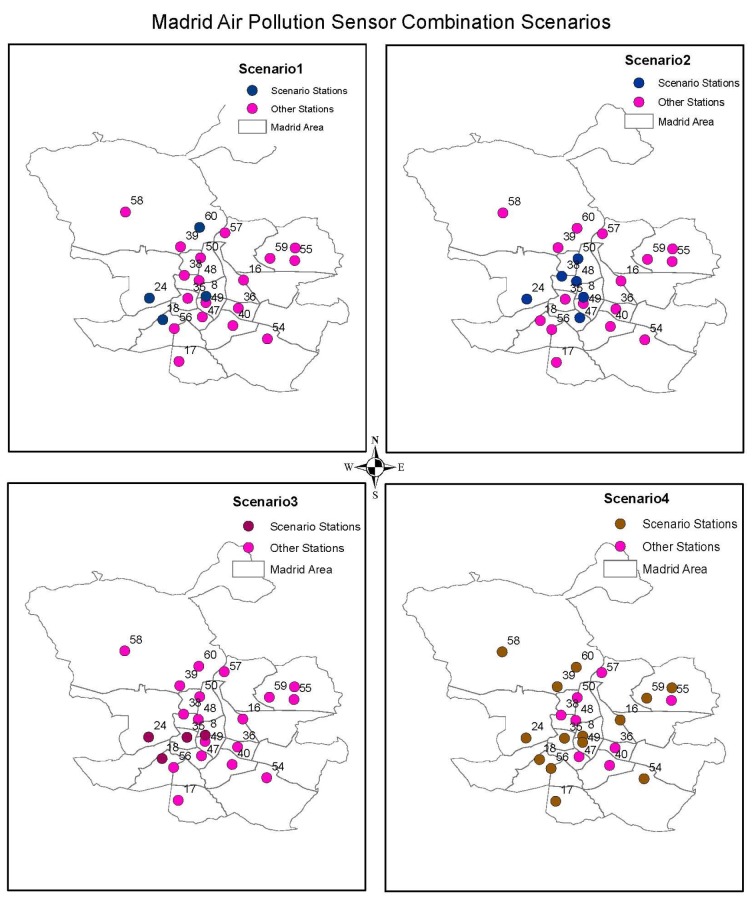
Madrid air quality stations combination scenarios.

We present the analyzed four scenarios in Figure 3, with maps showing the available stations for interpolation in each scenario.

Though the Madrid AQI shares some features with the UK DAQI, Ireland’s AQHI concerning the pollutants and the number of categories considered, there is a difference regarding reporting as the other two indices report the daily situation rather than an hourly situation reported by the Madrid AQI. Madrid AQI renders it challenging to compare the limit values of these indices.

The CAQI offers both hourly and daily indices but differs from the Madrid AQI regarding the number of categories and in their formulation. The CAQI is defined by five categories against four categories of Madrid AQI and has two types of AQI, the Roadside and Background AQIs. From the formulation of these indices and considering the hourly limit values, the limit values of NO${}_{2}$ and PM10 for the first two categories of CAQI for shallow and low are the same as that of the first category of Madrid AQI with a difference in O${}_{3}$ limits.

The Madrid AQI lacks PM2.5 in its formulation yet this pollutant is among the pollutants of concern in the city and thus the need for its inclusion in an AQI formulation.

### 4.4. Formulation of the Madrid Local Air Quality Index

From this study, a new AQI was suggested, the Madrid Local Air Quality Index (MLAQI), which was modified out of the used index in Madrid [22] and uses the CAQI’s idea of core and auxiliary pollutants [6]. The MLAQI is based on the categories and limit values of the Madrid AQI and CAQI. The pollutants considered in this index are NO${}_{2}$, O${}_{3}$, PM10 and PM2.5. The core pollutant for MLAQI is NO${}_{2}$, and the auxiliary pollutants are O${}_{3}$, PM10 and PM2.5. With MLAQI, the index at a given station should only be calculated with the existence of the core pollutant and at least one of O${}_{3}$ and PM10 pollutants. This is due to the inadequacy of PM2.5 measurements and the spatial distribution of the sensors for its measurements that would not represent the whole city while interpolated.

To get a sub index, compare a pollutant concentration with the defined limit values of that pollutant and the index range for this AQI as shown in the equation:

(1) $$S_{x}=\left(\left(P_{x}-P_{l}o\right)\right)/\left(\left(P_{u}p-P_{l}o\right)\right)*\left(I_{u}p-I_{l}o\right)+I_{l}o,$$

where $S_{x}$ is the sub-index, $P_{x}$ is the pollutant concentration measurement, $P_{l}o$ is the lower limit value for the range where the pollutant measurement falls, $P_{u}p$ is the upper limit value for the range where the pollutant measurement falls, $I_{u}pis$ the upper limit value of the index range and $I_{l}o$ is the lower index limit value for the range.

To get the final index at a given sensor station which qualifies for index calculation with MLAQI, use Equation (1) to calculate sub-indices of the available pollutants at that station. The final index is the highest of those sub-indices at that station. It is the index range which defines the category of the final index. The MLAQI is shown in Table 3 with the pollutant limit values used to calculate the sub-indices and the color coding for the respective categories.

**Figure 4:**
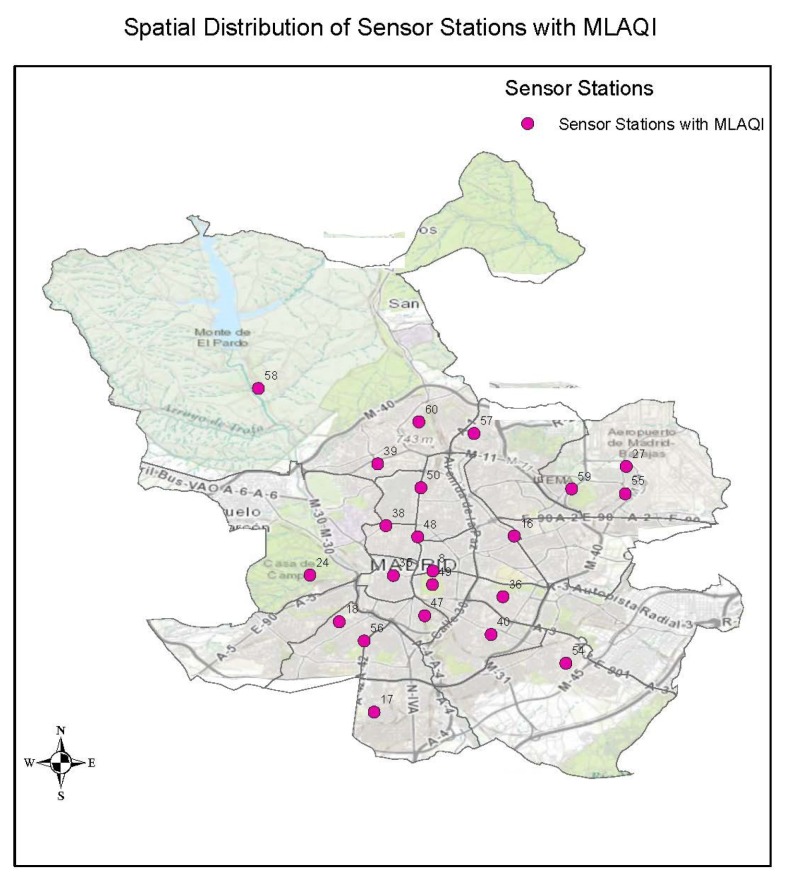
The spatial distribution of air quality stations with Madrid Local Air Quality Index (MLAQI).

The strengths of this index are that it considers the local situation of Madrid city, and it considers the composition of the available sensor network and the pollutants whose concentrations can be measured. With MLAQI, the sensor network facilitates 22 out of 24 sensor stations from which we can acquire data for interpolation to represent the air quality situation in Madrid. Figure 4 shows a map extract of the spatial distribution of the data with the defined and adopted index for the study.

To apply the defined AQI, the script detailed in previous subsections has been extended. When new observations are coming from the sensor network, the selected index is calculated in real time, obtaining a value for each station. These values will be used to interpolate and generate the final map layer in the next steps.

### 4.5. Applying a Suitable Spatial Interpolation

Before the choice of an interpolation method for this study, we have explored the available data to check for any errors, distribution and the existence of any outliers. Since the output of the interpolation was intended to serve a continuous interpolation process throughout the entire year for the real-time data published by the Madrid city council, we decided to test 2017 historical data for diurnal and seasonal consistency in its behavior. In the choice of time ranges for diurnal consistency analysis, we selected several hours of 7:00 a.m., 1:00 p.m. and 7:00 p.m. for a specific day on the 12th or 13th for several months. For seasonal consistency analysis, we chose January, April, July, and October, which are the middle months of every season. In total, 12 datasets have been created. We explored the data using the regional histogram and Voronoi polygons functionalities of ArcGIS software (10.6, ESRI, Redlands, CA, USA).

**Inverse Distance Weighting (IDW) structural modelling**: To analyze the data structure for modeling with IDW, we used six sets of different model parameters to analyze our 12 datasets. For each dataset, we first used the default parameters for modeling and recorded the model parameters together with their prediction errors.

**Variogram structural modelling**: To study the structure of our data for Kriging, we employed the use of the variogram and tried fitting two different models of spherical and exponential to get an optimum one to represent our phenomenon better. The exponential model appeared to fit better our phenomenon than the spherical model and was therefore used for further structural modeling of our datasets.

**Figure 5:**
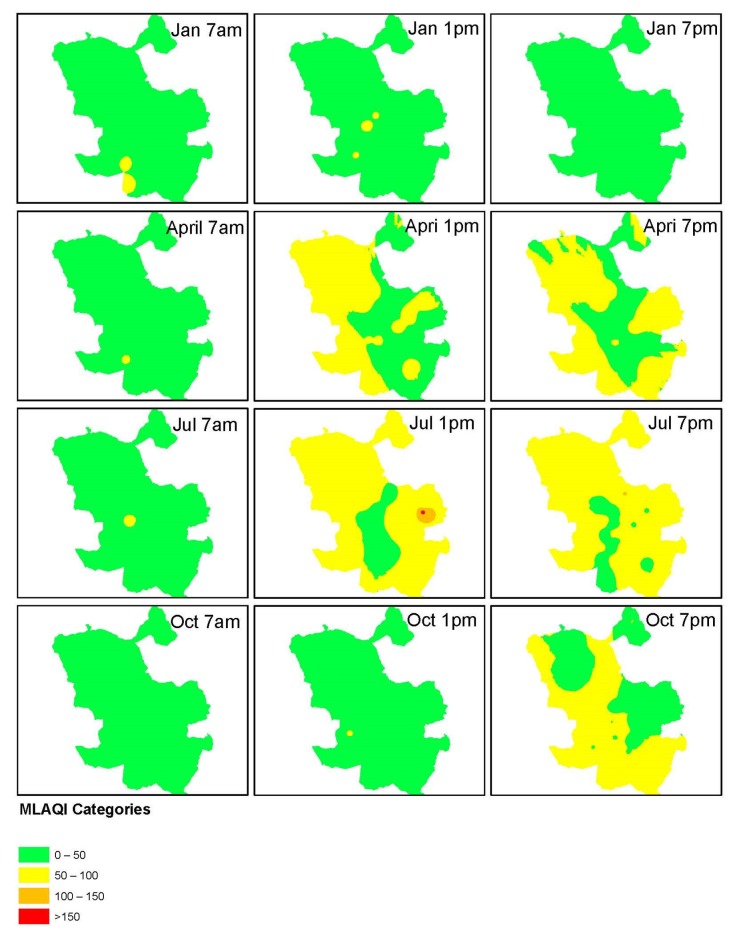
Categorised Inverse Distance Weighting (IDW) interpolation output according to MLAQI.

Though the results from both modelings, the IDW method fits better than Kriging in the defined use case. This decision is based on the IDW consistency in producing the highest frequency of 67% in obtaining the lowest magnitude root mean square prediction error. We categorized the output from IDW interpolation of all datasets according to the definition of MLAQI. We present the output from this process in Figure 5.

From Figure 5, the categorized surfaces mostly use the first two categories of MLAQI. The datasets for 7:00 a.m. data for all the months are mostly spread in the excellent category of MLAQI while those of 7:00 p.m. tend to spread in the acceptable categories. The great category of MLAQI mostly depicts the data from January. The acceptable category more represents the data from April and July (2017) for the afternoon hours.

The main script based on ArcGIS is in charge of executing the selected interpolation model when new data from Madrid sensor networks arrive.

### 4.6. Map Processing Service

The input to this process is the generated layer from the IDW interpolation provided by the main script. The objective is to create a map layer based on the results of the interpolation model. To achieve this goal, a new module (in the main script) is created based on the ArcGIS server as well.

The process uses the *GaLayerToContour* tool using the filled contour type with the class breaks of the MLAQI index to generate an index categorized polygon feature class. The extent environment settings for this process are set using the extent of the Madrid boundary polygon feature class. It is used to extend the processing environment outside the location of the sensor locations’ point data to generate a representation for the entire area of Madrid.

Using the clip analysis tool, the generated polygon feature class is now clipped with the Madrid boundary polygon feature class to keep it within the shape extent of the study area. The output from the clipping is projected to a WGS1984 Web Mercator Auxiliary Sphere coordinate system to support the drawing of the features on the web.

Using the select analysis tool, the projected polygon feature class is separated into four polygon feature classes according to the MLAQI index. When the polygons are generated, the next step is to publish them as web map layer to share the results online.

### 4.7. Routing Service to Avoid Pollution Zones

When the web map layer is published, the calculated areas can be used as input barriers when generating routes across a city. An ArcGIS Online network analysis service is used for this purpose [23]. This routing service models driving and walking transportation modes. It can be set up to calculate shortest time or distance route from points A to B. The analysis of this routing service takes into consideration traffic flow directions and turn restrictions.

When a highly polluted area is used as an input barrier in the routing service, it excludes all intersecting streets from the analysis and calculates a pollution free route. Madrid and its metropolitan area are entirely covered by this routing service hosted and managed by Environmental Systems Research Institute (ESRI’s) ArcGIS Online platform.

### 4.8. A Web Client Able to Trace Pollutant-Free Routes

With the created web map service and routing service as inputs, we created a web-application based on the ArcGIS API for JavaScript is developed, it mixes different technologies, such as HTML5, JavaScript or Cascading Style Sheets (CSS). It allows the capacity for building a responsive client, as it can adapt to the device’s features (desktop, mobile or wearable).

The web-application queries the feature layers from the WFS service in a avaScript Object Notation (JSON) format using the query module, checks if those layers contain features in them and adds such features to the route parameters as polygon barriers. These polygon barriers are used to limit the user navigation in these high pollution areas. A route generated with a polygon barrier between the locations of interest will minimize the traveling cost parameter and avoid these areas with high pollution index categories.

The routing task uses the routing layer and defines the route parameters such as stops, polygon barriers, impedance attribute name and spatial reference to determine the route. The used travel impedance for the study is kilometres, thus the resulting shortest route.

Some improvements have been deployed to increase the user’s interaction. These include the clear stops and route button to enable the user route result and perform another route task. The search bar adds the functionality to find a location of interest, a geolocation button to enable the user to locate their position on the map. In addition, the web-application has the zoom and home buttons for map navigation, the legend for identifying index categories, and the base map toggle tool to enable the different visualizations.

**Figure 6:**
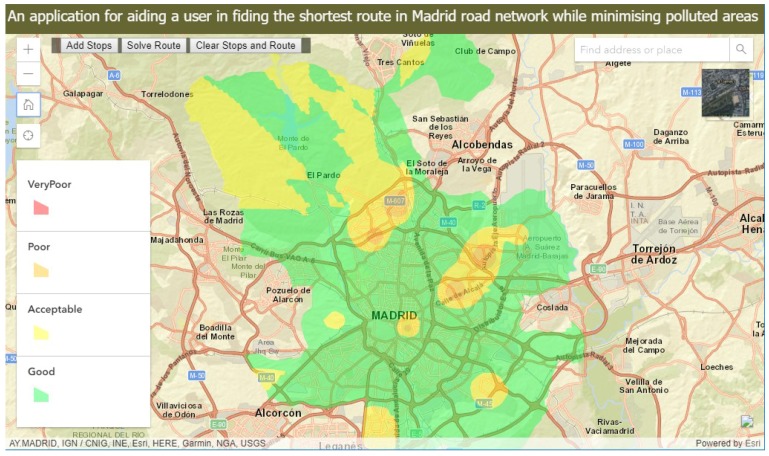
A screenshot of the developed application.

The final developed web-application is published at [23]. A screenshot of the web-application is shown in Figure 6.

**Figure 7:**
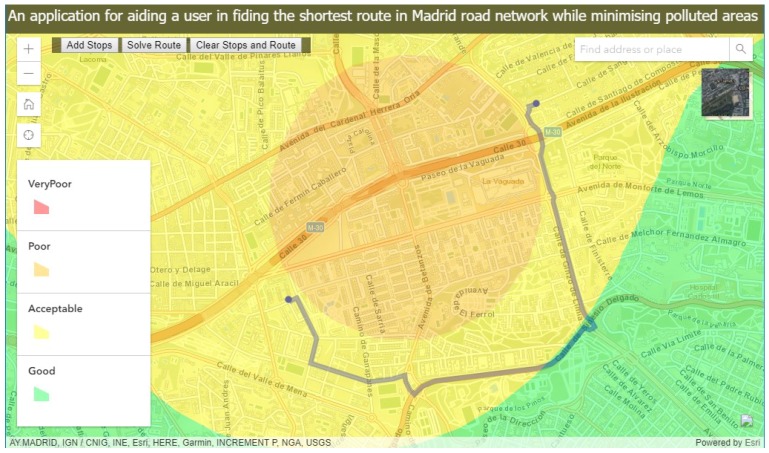
A route with minimised polluted areas.

To protect the user’s health against the polluted area of poor and very poor categories, the application offers the user with functionality to solve a route for navigation through a Madrid road network that minimizes these polluted areas. A screenshot of such a route is shown in Figure 7.

## 5. Conclusions

The presented work proposes a technology-agnostic methodology to trace pollution-free routes in real time across cities using air quality sensor networks. To achieve this goal, some previous studies have been detailed in Section 2. In addition, a review of some AQI has been shown to choose the most suitable index for the selected use case. Finally, Section 4 presents a use case in Madrid and takes Madrid’s air quality sensor network to develop and validate the conceptual components detailed in Section 3 to trace pollution-free routes in real time.

To apply the methodology to Madrid’s use case, a modification of the existing MAQI for better air pollution representation with the support of spatial interpolation proposed. To support the dissemination of these results to smart citizens and improve their health, there is a need for creating a real-time conversion service to generate vector geometries from the interpolated raster surfaces into categories of Good, Acceptable, Poor and Very Poor according to the index. Finally, a web-application using the published service has been created to plot routes by minimizing the high pollution areas to traverse, a way to improve their health.

The proposed methodology provides a platform to help in building services aimed at helping the citizens be aware of the air quality around them. However, other stakeholders can use it to develop third-party applications. For instance, using the final output (map service) can help public administrations to make informed decisions while planning their activities. The final result can be used by other developers as a data source to create other applications aimed at public awareness of the air pollution around them in real time.

From this study, an observation was made that data will not always be in a ready geospatial format for individual studies, but geospatial technologies are enablers to extract and format such data to serve the purpose of such studies.

In the literature, there are not a lot of related works, but some approaches use air quality data to know the exposure level in routes [24,25]. Ref. [24] offers routes with the best value of PM2.5 exposure during a route. There are some studies where sensor nodes are deployed in buses [25] or cars [26,27].

Any work that listed works with AQI, different from ours, only considers unique phenomena. Our work, as we detailed, previously proposed AQI considering different phenomena. Another difference from previous related works is that some of them are installing sensors on vehicles to know the level in streets for cars. Our work is using official stations to create an interpolated surface, and we can trace routes for pedestrians, cyclists or any vehicle.

The following items describe the works that have been analyzed. All of them talk about IoT in the smart agriculture context, more concretely on monitoring vineyards.

The work detects two limitations, the first one is the number of active sensor stations in the sensor network, in this case, the Madrid network. It may affect the accuracy of spatial interpolation. The second limitation is the ESRI routing service because it limits the number of the intersected street with polygon barriers in routing.

As a future work, with the challenge of varying values during the variogram modeling, further research could be performed with more spatiotemporal analysis of the hourly behavior of the pollution situation in Madrid. This would help us have a better understanding about episode hours in Madrid and whether hourly different interpolation model parameters could apply to achieve better interpolation. The interpolation could be supplemented with other parameters like the elevation of sensor stations to test the variability of pollutant concentration measurements over different elevation. A routing application could be extended into a mobile application and try to render the interpolation layer as 3D surfaces, in order to take better awareness about the pollution in real time inside cities by citizens.

## Figures and Tables

**Table 1:** Summary of reviewed indices from government bodies and the research community.

Index	Pollutants Considered	Number of Categories	Ranges of Categories	Symbolisation	General Health Recommendations	Specific Groups Recommendations	Multi-Pollutant Consideration	Measurement Location Variation
US EPA, AQI	CO, NO${}_{2}$, O${}_{3}$, PM 2.5, PM 10, SO${}_{2}$	6	0–500	Colours	✓	✓	✗	✗
Canada, AQHI	O${}_{3}$, NO${}_{2}$, PM 2.5	4	1–10+	Colours	✓	✓	✓	✗
Common Air Quality Index, CAQI	CO, NO${}_{2}$, O${}_{3}$, PM 2.5, PM 10, SO${}_{2}$	5	0–100+	Colours	✗	✗	✗	✓
UK Defra, DAQI	NO${}_{2}$, O${}_{3}$, PM 2.5, PM 10, SO${}_{2}$	4	1–10	Colours	✓	✓	✗	✗
Irish EPA, AQIH	NO${}_{2}$, O${}_{3}$, PM 2.5, PM 10, SO${}_{2}$	4	1–10	Colours	✓	✓	✗	✗
Spain Madrid	CO, NO${}_{2}$, O${}_{3}$, PM 10, SO${}_{2}$	4	0–>150	Colours	✗	✗	✗	✗
France, ATMO	NO${}_{2}$, O${}_{3}$, PM 2.5, PM 10, SO${}_{2}$	3	1–10	Giraffe and Colours	✗	✗	✗	✗
Singapore, PSI	CO, NO${}_{2}$, O${}_{3}$, PM 2.5, PM 10, SO${}_{2}$	5	0–500	Colours	✓	✓	✗	✗
Cairncross et al. 2007, API	CO, NO${}_{2}$, O${}_{3}$, PM 2.5, PM 10, SO${}_{2}$	4	1–10	Colours	✓	✗	✓	✗
Stieb et al. 2008, AQHI	NO${}_{2}$, O${}_{3}$, PM 2.5 or PM 10	4	1–10+	Colours	✓	✓	✓	✗
Kyrkilis et al. 2007, Aggregate AQI	CO, NO${}_{2}$, O${}_{3}$, PM 10, SO${}_{2}$	-	-	-	-	-	✓	✗
Sicard et al. 2011, ARI	NO${}_{2}$, O${}_{3}$, PM 2.5, PM 10, SO${}_{2}$	4	0–10	Colours	✓	✓	✓	✗
Murena 2004, PI	CO, NO${}_{2}$, O${}_{3}$, PM 10, SO${}_{2}$	5	0–100	Clouds	✗	✗	✓	✓

**Table 2:** Comparison of Air Quality Index (AQI) and the available pollutants.

Index	Pollutant Combinations						Sensor Stations
AQHI (Canada)	-	Ozone	Nitrogen dioxide	-	-	PM2.5	2
CAQI (Roadside)	Carbon monoxide		Nitrogen dioxide	-	PM10	PM2.5	2
CAQI (Background)	Carbon monoxide	Ozone	Nitrogen dioxide	Sufur Dioxide	PM10	PM2.5	2
DAQI (UK Defra)	-	Ozone	Nitrogen dioxide	Sufur Dioxide	PM10	PM2.5	2
Spain Madrid	Carbon monoxide	Ozone	Nitrogen dioxide	Sufur Dioxide	PM10	-	3
ATMO (France)	-	Ozone	Nitrogen dioxide	Sufur Dioxide	PM10	PM2.5	2

**Table 3:** Madrid Local Air Quality Index definition.

Index Range	Index Category	Color	Core Pollutant	Auxiliary Pollutant		
			NO${}_{2}$	O${}_{3}$	PM10	PM2.5
0–50	Good	Green	0–100	0–90	0–50	0–30
51–100	Acceptable	Yellow	101–200	91–180	51–90	31–55
101–150	Poor	Orange	201–300	181–240	91–150	56–90
>150	Very Poor	Red	>300	>240	>150	>90
